# Narrative review of application of metagenomic approaches to study the link between oropharyngeal microbiome and infectious diseases

**DOI:** 10.3389/fmicb.2023.1292526

**Published:** 2023-12-07

**Authors:** Kanny Diallo, Kouassi Firmin Missa, Jeremie Kolotioloman Tuo, Tiemele Laurent Simon Amoikon, Brice K. Bla, Bassirou Bonfoh

**Affiliations:** ^1^Centre Suisse de Recherches Scientifiques en Côte d’Ivoire (CSRS), Abidjan, Côte d’Ivoire; ^2^West African Centre for Cell Biology of Infectious Pathogens (WACCBIP), University of Ghana, Accra, Ghana; ^3^Université Félix Houphouët Boigny de Cocody, Abidjan, Côte d’Ivoire; ^4^Institut National Polytechnique Félix Houphouët-Boigny (INP-HB), Yamoussoukro, Côte d’Ivoire

**Keywords:** oropharyngeal microbiome, next generation sequencing, biomarkers, metagenomic, infectious disease

## Abstract

**Context:**

Viral and bacterial infections are major causes of morbidity and mortality worldwide. The oropharyngeal microbiome could play an important role in preventing invasion of viral and bacterial pathogens by modulating its content and the host’s innate immune response. Next Generation Sequencing (NGS) technologies now enable in-depth study of the genomes of microbial communities. The objective of this review is to highlight how metagenomics has contributed to establish links between changes in the oropharyngeal microbiome and emergence of bacterial and viral diseases.

**Method:**

Two search engines, PubMed and Google scholar were used with filters to focus searches on peer-reviewed original articles published between January 2010 and September 2022. Different keywords were used and only articles with metagenomic approaches were included.

**Results:**

This review shows that there were few articles studying the link between oropharyngeal microbiome and infectious diseases. Studies on viruses using metagenomic techniques have been growing exponentially in recent years due to the Covid-19 pandemic. This review shows that most studies still focus on the basic identification of microorganisms in different disease states and multiple microorganisms (*Alloprevotella, Prevotella, Bacteroides, Haemophilus, Streptococcus, Klebsiella* sp.*, Acinetobacter sp…*), have been associated with development of infections such as childhood wheezing, *influenza*, Covid-19, *pneumonia*, *meningitis*, and *tuberculosis*.

**Conclusion:**

The oropharyngeal microbiome, despite its importance, remains poorly studied. A limited number of articles were identified but this number has increased exponentially since 2020 due to research conducted on Covid-19. These studies have shown that metagenomic has contributed to the unbiased identification of bacteria that could be used as biomarkers of various diseases and that further research is now needed to capitalize on those findings for human health benefit.

## Introduction

The first methods to study microorganisms were based on culture, morphological and biochemical analysis. These methods limited the vision of the microbial world, before the development of molecular techniques such as Sanger sequencing (Sanger et al., 1977). Other techniques such as cloning, fluorescence *in situ* hybridization (FISH) and use of molecular markers have also contributed to a better understanding of microbial content of various type of samples (Schramm et al., 2002).

Despite being an important technique, limitations are associated with culture-based methods for characterizing microbial communities. They are labor intensive, time consuming and sometimes costly or risky for the technician. Additionally, it is still difficult to grow large number of microorganisms in laboratory conditions, and biochemical classification can be misleading (Maleki et al., 2020).

Metagenomic was developed to study microbial biodiversity of any environment without the need to grow and isolate pathogens. This recent technique rely on the unbiased extraction of genetic material present in samples and identification of microorganisms through sequence-based technologies.

### Amplicon based metagenomic

#### 16S rRNA sequencing

Amplicon sequencing technology based on 16S rRNA sequencing was first introduced by ecologists in the 1980s (Proctor et al., 2019). Biomedical researchers then adopted this approach to explore the microbial diversity of the human body. The method is based on sequencing of one or more of the nine hypervariable regions (V1 to V9) of the ribosomal RNA (16S rRNA) gene (Paster et al., 2001; Aas et al., 2005). That gene is ubiquitously present in all existing bacteria. The 16S rRNA variable regions vary from one genus to the other but are flanked by conserved sequences in all bacteria. This has enabled PCR based amplification and sequencing of the variable regions which can be used to identify bacterial species present in samples through bioinformatic analyses.

16S rRNA sequencing, has changed the understanding of microbial diversity and has been used to analysis various type of samples from the environment (Tringe and Rubin, 2005) and health fields (Clemente et al., 2012). However, sequence diversity of the variable regions often give poor resolution, especially at the species level (Kai et al., 2019).

The sensitivity of different 16S rRNA variable regions to detect different bacterial communities can vary making comparison among microbiome studies often difficult. Some regions provide better identification than others (Bukin et al., 2019); for example, V1-V3 regions can discriminate between staphylococcal populations (Conlan et al., 2012), whereas V3-V4 were shown to be better at discriminating a greater number of taxa in the vaginal microbiota such as *Gardnerella vaginalis, Bifidobacterium bifidum*, and *Chlamydia trachomatis* Graspeuntner et al. (2018). 16S rRNA-based techniques can also be limited by high risk of contaminations during the processing of samples, sequencing errors, and difficulties in interpreting the presence of different operational taxonomic units (OTUs) (Quince et al., 2009, 2011; Youssef et al., 2009).

The first metagenomic studies focused on health targeted mainly the gastrointestinal tract and revealed significant metabolic potential for the human gut microbiota, including regulation of host fat storage, maintenance of mucosal immune homeostasis, and host-microbial symbiosis (Eckburg et al., 2005; Li et al., 2019).

#### Other genes targeted for amplicon based metagenomic

Although 16S rRNA remains the most used target, other genes have been explored to improve the resolution of metagenomic analyses. These are often tested and validated for a specific family or genus: for example, *amoA* has been used for metagenomic analysis of archaea (Bartossek et al., 2012; Wang et al., 2021); *porA* has also been used for the analysis of campylobacter species (Colles et al., 2019); and other genes are being explored as potential targets for metagenomic analysis.

### Whole genome metagenomic

Whole genome metagenomic sequencing (WGmS) does not focus on a single gene but rather on all the genetic material present in a sample. This advanced method relies on heavy bioinformatic analysis to reconstitute the genomes of the different organisms present in a sample. Although quite expensive, WGmS is becoming more used especially in laboratories that study complex samples which contain many unculturable microorganisms (Mardis, 2011).

One of the major difficulties encountered with samples coming from human health related studies has been the over-representation of human DNA in the samples to be analyzed. However, protocols that deplete human DNA have been elaborated to ensure more efficient targeted sequencing of the different microbes DNA (Horz et al., 2010; Jervis-Bardy et al., 2015; Nelson et al., 2019).

### Metagenomic application in health research

The human body, including the gut, skin mucosa, respiratory tract, urogenital tract, and mammary gland, is colonized by a considerable number of microorganisms, collectively called microbiota (Danping Zheng, 2020). The collective genomes of symbiotic and pathogenic bacteria and other microorganisms in this ecosystem, including fungi, viruses, and parasites, are called the microbiome (Berg et al., 2020) and have been recognized as an important part of the human body. As a result, it has been increasingly studied over the past two decades, thanks to the rapid development of genomic sequencing techniques described above.

The emergence of new sequencing technologies and bioinformatics pipelines, has led to a shift in clinical microbiology and infectious disease due to the realization of the complex interactions that occur within the microbiome (Malla et al., 2019).

The human microbiota is considered beneficial to the host by promoting differentiation of mucosal structure and function, stimulating the innate and adaptive immune systems, and providing “colonization resistance” against pathogen invasion (Yi et al., 2014). Colonization by microorganisms begins immediately after birth and is followed by the gradual assembly of species into a complex and dynamic microbial community (Biesbroek et al., 2014). Studies have shown that during the first week of life, niche differentiation in the upper respiratory tract leads to a high abundance of *Staphylococcus* spp. followed by enrichment of *Corynebacterium* spp. and *Dolosigranulum* spp. and subsequent dominance of *Moraxella* spp. (Bosch et al., 2016).

It has been shown that the human pharyngeal microbiome, which lies at the junction of the digestive and respiratory tracts, can play an active role in preventing respiratory infections, similar to the actions of the gut microbiome (Gao et al., 2014). Indeed, during healthy state, the microbiome is assumed to be in balance; containing various amount of mostly six phyla: *Actinobacteria, Bacteroidetes, Cyanobacteria*, *Firmicutes, Fusobacteria* and *Proteobacteria* (Danping Zheng, 2020).

In healthy adults, the nasopharynx is colonized by the genera *Streptococcus, Haemophilus, Neisseria* spp. and Gram-negative anaerobic species such as *Veillonella, Prevotella, Leptotrichia* and *Fusobacterium*. Several pathogens of the genus *Streptococcus* are also present in the pharynx such as *S. pneumoniae* and *S. pyogenes*. These can cause serious disorders not limited to the pharynx, and can also cause septic shock (Kumpitsch et al., 2019; Santacroce et al., 2020). This balance state, which can vary from one individual to the other, represent the normal composition of the microbiota and provides beneficial functions to the host such as resistance to pathogens (Cho and Blaser, 2012). However, changes in the composition of the microbiota, acquisition of new bacterial or viral pathogens, environmental factors, or immunological perturbations can potentially disrupt this balance, leading to dysbiosis, proliferation, and spread of pathogens, and to symptomatic infections such as pneumonia, cystic fibrosis, and even Covid-19 (de Koff et al., 2016; de Steenhuijsen Piters et al., 2016; Xiang et al., 2020).

Most microbiome studies have focused on the gut microbiota, which has even been considered an “essential organ,” carrying about 150 times more genes than the entire human genome (Wang et al., 2017). This microbiome plays a prominent role in human health by influencing the development of chronic diseases ranging from metabolic diseases to gastrointestinal disorders (Ogunrinola et al., 2020).

Relatively less effort had been made to understand the microbiome in other parts of the body; this has been confirmed by the disproportionate number of peer-reviewed articles addressing the gut microbiome compared to other body sites, at the time of writing. However, some studies have also worked on the oral microbiome, primarily on periodontally associated conditions such as dental caries, oral, and esophageal cancer (Willis and Gabaldón, 2020).

The purpose of this review is to highlight studies of the oral microbiome linked to invasive diseases other than those associated with periodontal disease, in order to establish the relationship between changes in the oropharyngeal microbiome and bacterial and viral diseases. These diseases caused by viruses (i.e., Covid-19) and bacteria (i.e., *Streptococcus pneumonia*, *Neisseria meningitidis*…), are important global public health threats and have been responsible for epidemics that are major causes of morbidity and mortality in the world. The overall objective is to identify new research avenues for development of preventive measures via modulation of the oropharyngeal microbiota; as done for the gut microbiota. Understanding the link between the microbiome and this type of diseases is crucial to the ability to develop clinical applications to prevent these infections; alongside other measures such as vaccination.

### Methodology

Peer-reviewed articles and literature reviews were targeted using Google scholar and PubMed search engines; however, relevant preprints were included for Covid-19.

This review includes articles published between January 1, 2010 and September 20, 2022. Search terms included “oropharyngeal microbiome and bacterial diseases,” “oropharyngeal microbiome and viral diseases.” Individual articles from each search engine were first filtered based on titles and abstracts: any studies focusing on microbiomes of the gut or other body sites and periodontal diseases were removed from the list. Then, the filtered lists recovered for each search engine were merged and duplicates were eliminated. The remaining articles were then further analyzed to identify the ones which used a metagenomic approach (16S or others). These selected articles were included and analyzed. The articles were organized using Excel software.

**Figure 1:**
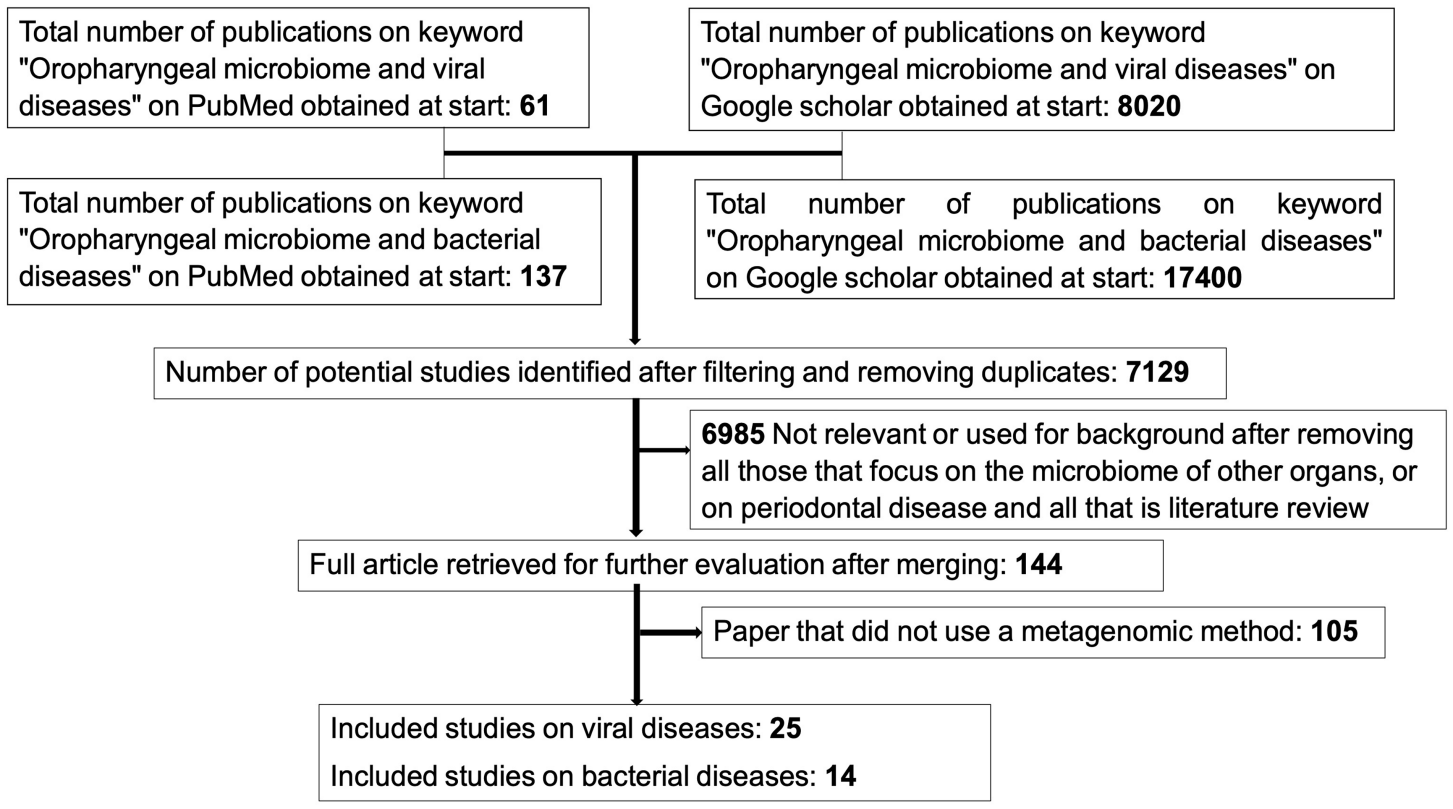
Literature review flowchart.

A total of 198 articles were identified on PubMed and 25,420 articles on Google scholar. We first found 61 publications with the keyword “Oropharyngeal microbiome and viral diseases” and 137 publications with the keyword “Oropharyngeal microbiome and bacterial diseases” on PubMed. Similarly, 8,020 publications with keyword “Oropharyngeal microbiome and viral diseases” and 17,400 publications with keyword “Oropharyngeal microbiome and bacterial diseases” were found on Google scholar. After filtering and eliminating irrelevant articles based on titles, abstracts and methodologies used, and removing duplicates from the different search engines, we retained 25 studies on viral diseases and 14 studies on bacterial diseases (Figure 1).

## Results

The majority of the articles obtained in this literature review focused on viral infections such as influenza, Covid-19, HIV, *respiratory syncytial virus* infections. For bacterial infection, they focus of pneumonia, meningitis, tuberculosis and some pharyngeal sexually transmitted infections.

**Figure 2:**
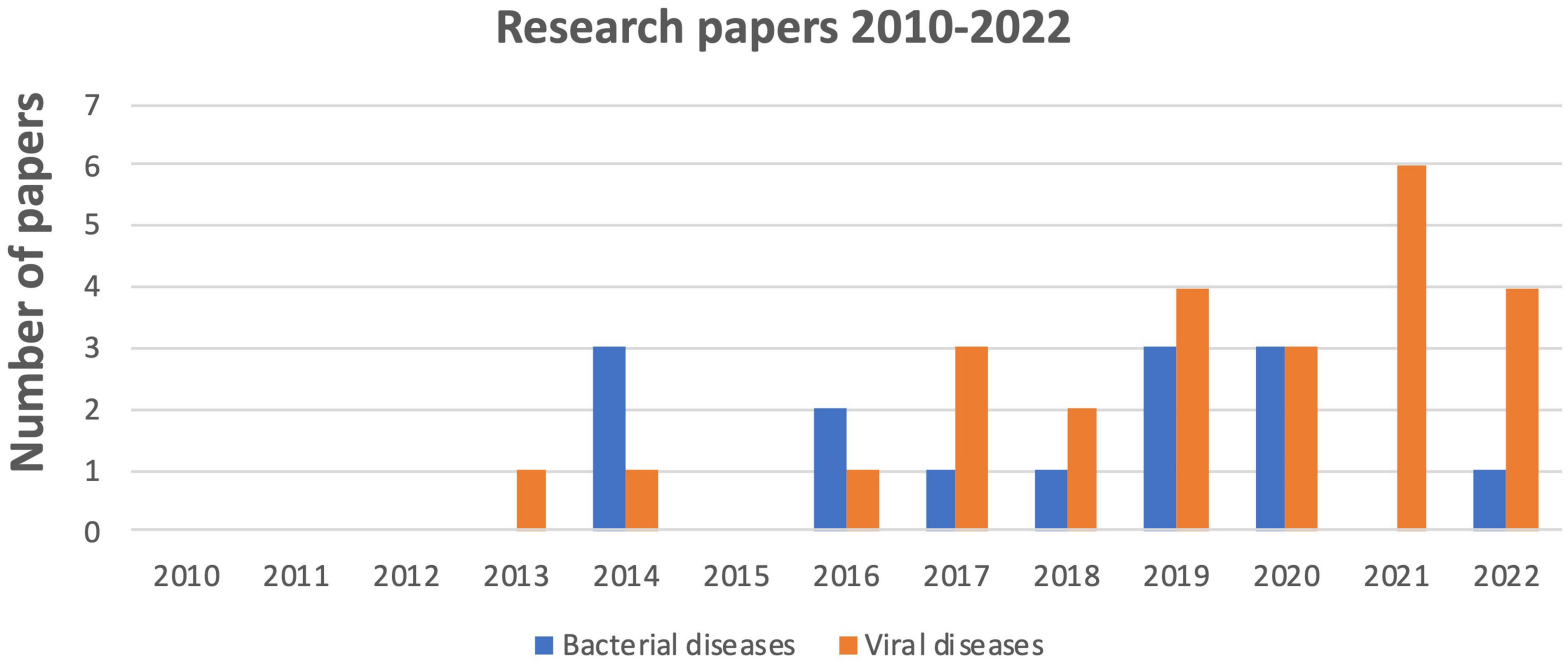
Evolution of the number of publications on the oropharyngeal microbiome linked to bacterial and viral disease from 2010 to 2022.

The number of articles considered in this review has gradually increased over time, with a significant increase in 2021 due to studies associated with viral diseases, focusing mainly on Covid-19, unlike bacterial infections for which we found no relevant publication in 2021 (Figure 2).

### Oropharyngeal microbiome in viral diseases

#### Cytomegalovirus

The viral microbiome or virome is the collective genomes of all viral microorganisms. The viral community supplies a pool of mobile genetic information that can give novel functional properties to surrounding pathogens. To study the viral microbiome in healthy individuals, Thannesberger et al. (2017) collected oropharyngeal lavages, urine, and fecal samples from healthy individuals and four patients with primary cytomegalovirus infection for evaluation of sensitivity and reproducibility of the preanalytic procedure of virus isolation. The QIAamp Virome RNA/DNA kit was used to enrich virions and isolate viral nucleic acids, and contaminant 18S rDNA and 16S rDNA were detected with use of specific PCR assays. The oropharyngeal and urinary virome samples were evaluated using Illumina MiSeq platform sequencing. As results, the majority of putative phage sequences were assigned in the oropharyngeal and urinary samples (97 and 81%, respectively) to *chlamydiamicrovirus* that infects *Chlamydia* spp. The majority of nonphage sequences were assigned in both samples to *Anelloviridae* (65 and 68%, respectively). During acute eukaryotic virus infection in patients with primary *cytomegalovirus* infection, the virus is excreted in urine and the oropharynx for prolonged periods and was proposed to modify the susceptibility to a range of other infectious pathogens. *Cytomegalovirus* DNA was undetectable in the oropharyngeal and urinary samples, despite use of a highly sensitive, virus-specific quantitative PCR assay. The abundance of hits to phage markers was highly variable among the different samples and ranged from 29 to 99% in urinary samples and from 47 to 99% in oropharyngeal samples. In contrast, sequence similarity with known phage markers was lower overall in oropharyngeal samples, which was indicative of an abundance of novel phage. The overall observation of this study was that the discovery of new viral sequences was more related to the intrinsic factors of the individual being evaluated than the ecological niche probed (Thannesberger et al., 2017).

#### Acute viral wheeze

The development of asthma can be a consequence of viral acute wheezing, which is the major cause of hospitalization in children. Severe wheezing episodes in children could be associated with bacterial dysbiosis in the airways (Cuthbertson et al., 2019). Two studies compared oropharyngeal samples using 16S rRNA sequencing with the Illumina MiSeq platform in the development of recurrent wheezing. Cuthbertson et al. (2019) compared wheezing children of tertiary pediatric hospital in Perth, Australia with samples from non-whistling controls and showed no significant difference in bacterial diversity between samples from wheezing individuals and healthy controls. Wheezing and viral infection were therefore not significantly related to the bacterial community in this study. According to the study, the increase of bacterial diversity in the wheezing group was associated with kindergarten or nursery school attendance. Likewise, the oropharyngeal bacterial microbiome was highly variable in early life and its role in wheezing remains less clear than viral influences (Cuthbertson et al., 2019). Zhang et al. (2020) conducted another study on the possible association between respiratory microbiome, host immune response, and the development of recurrent wheezing in infants with severe *respiratory syncytial virus* (RSV) bronchiolitis from Beijing Children’s Hospital, China. They found that the relative abundance of *Haemophilus*, *Moraxella*, and *Klebsiella* was higher in infants who later developed recurrent wheezing than in those who did not. Airway levels of lipopolysaccharides (LPS) were also significantly higher in infants who later developed recurrent wheezing than in those who did not. Moreover, high airway abundance of *Haemophilus* was associated with increased level of the chemokine CXCL8, and that of *Moraxella* was associated with increased level of cytokines IL-6 and IL-10 that are markers of immune responses. This study suggests that higher abundance of *Haemophilus* and *Moraxella* in the airway might modulate local inflammation during severe RSV bronchiolitis in infancy, potentially contributing to the development of subsequent recurrent wheezing in later childhood (Zhang et al., 2020).

#### Influenza

Influenza is a major cause of morbidity in low- and middle-income countries. Many studies indicate that the upper respiratory microbiota plays a critical role in respiratory health and that dysfunctional respiratory microbiota leads to disease. Despite this, the impact of the microbiota during influenza is under-researched (Wen et al., 2018). The majority of the studies found have focused on the impact of influenza on the upper respiratory microbiome. A study conducted by Lee et al. (2019) on the home transmission of influenza showed that nose and throat microbiota were associated with susceptibility to influenza in participants exposed to influenza virus in their homes. Using 16S (V4) rRNA sequencing and a generalized linear mixed effects model, they found a type of nasal/oropharyngeal microbiota associated with decreased susceptibility to influenza. This nasal/oropharyngeal community type was rare and transient in young children but prevalent and stable in adults. Using linear boosting and mixed effects models an association between nose and throat microbiota and influenza was found at the taxon level, specifically with the relative abundance of *Alloprevotella*, *Prevotella*, and *Bacteroides oligotypes*. A high diversity rate was observed between bacterial microbiota in both secondary cases and household contacts who were not infected during follow-up. Although further studies are needed to determine causality, these results suggest that the nose and throat microbiome may be a potential target for reducing the burden of influenza (Lee et al., 2019). Moreover, another study of household transmission of influenza virus showed that a 10-fold increase in the abundance of *Streptococcus* spp. and *Prevotella salivae* was associated with a 48% decrease in susceptibility to *influenza A* (H3N2) infection. A 10-fold increase in the abundance of *Streptococcus vestibularis* and *Prevotella* spp. was associated with 63% reduction and 83% increase in influenza *B* infection, respectively. If these associations are proved to be causal, it would suggest that the microbiome could be modulated to reduce the risk of influenza infection (Tsang et al., 2020). Wen et al. (2018) conducted a study, of the nasopharyngeal and oropharyngeal microbiota of children with *influenza A virus* compared to healthy controls using 16S rDNA sequencing and compared microbiota structures in different individual at Shenzhen children’s hospital, China. The dominant genera in the nasopharynx of sick patients, such as *Moraxella*, *Staphylococcus*, *Corynebacterium*, and *Dolosigranulum* were less abundant in healthy children. The genera *Streptococcus* and *Phyllobacterium* were significantly more present in the nasopharyngeal microbiota of patients while the most abundant genera in the oropharyngeal microbiota including *Streptococcus*, *Neisseria*, and *Haemophilus* showed a decreasing trend in patients (Wen et al., 2018). Influenza virus and *Mycoplasma pneumoniae* (MP) are two pathogens that frequently cause acute respiratory tract infections in pediatrics. Zhou et al. (2020) compared the acute airways of patients from Shenzhen Children’s Hospital, China with *MP pneumonia*, patients with influenza virus infection and healthy children. They found that the microbial diversity of oropharyngeal and nasopharyngeal samples decreased in pediatric *Mycoplasma pneumoniae* infection and increased in influenza infection compared to healthy children. *Staphylococcus* species dominated the nasopharyngeal microbiota of patients with *Mycoplasma pneumoniae pneumonia* (MPP), while *Streptococcus* was significantly enriched in the MPP group and decreased in the Influenza group. Decision tree analysis indicated that *Ralstonia* and *Acidobacteria* could discriminate microbial samples in the healthy, MP and Influenza groups with high accuracy. This study let out to the conclusion that the preponderant bacterial structure in the airways depend on the environment and disease specificity (Zhou et al., 2020).

Similarly, to understand the impact of influenza on the upper respiratory microbiome, Ramos-Sevillano et al. (2019) examined oropharyngeal samples from volunteers in a Covid-19 quarantine facility in the United Kingdom taken over a 30-day period after experimental intranasal inoculation of 52 healthy adult volunteers with *influenza A*; comparing them to samples obtained from 35 healthy control subjects. The V1-V3 region of 16S ribosomal RNA were amplified and sequenced using a Roche 454 GS-FLX Titanium platform to determine the composition of the microbiome (Ramos-Sevillano et al., 2019). A total of 43 of the 52 volunteers developed proven influenza infections, of which 33 became symptomatic. None of the controls developed influenza, even though 22% reported symptoms and no significant difference was observed in the microbiota over time between individuals with influenza and those in the control group, the diversity of bacterial communities remained unchanged after the acquisition of influenza. There was no change in colonization rates by *Streptococcus pneumoniae* or *Neisseria meningitidis*. These results indicate that the throat microbiota was resilient to influenza infection in that study (Ramos-Sevillano et al., 2019).

Leung et al. (2013) recruited pneumonia patients with and without pH1N1 infection and characterized their oropharyngeal microbiota by whole genome metagenomic sequencing using the Illumina Solexa Sequencing Platform of the Beijing Genome Institute Shenzhen. *Pseudomonas* species with chemotaxis and flagellar assembly genes increased significantly (>20-fold) in the pH1N-infected group while *Bacillus* and *Ralstonia* species that also increased significantly (5–10 fold) had similar signaling and motility genes. The abundance of oral commensal species *Prevotella*, *Veillonella*, and *Neisseria* decreased significantly during pH1N1 infection. These results suggest that pH1N1 infection may promote an environment conducive to respiratory pathogens gaining access to the lower respiratory tract of people with low immunity (Leung et al., 2013).

We found four studies that examined the oropharyngeal and nasopharyngeal microbiome in influenza transmission, three studies used 16S rRNA sequencing but targeted different regions the V1-V3 and V4 whereas one employed whole genome metagenomic sequencing to study the microorganism’s community. The associations between oropharyngeal, nasopharyngeal microbiome and influenza existed at the taxon level, specifically with the relative abundance of *Alloprevotella*, *Prevotella*, and *Bacteroides oligotypes.* The dominant genera such as *Moraxella*, *Staphylococcus*, *Corynebacterium*, and *Dolosigranulum Streptococcus*, *Neisseria* and *Haemophilus* in the nasopharynx of sick patients were less abundant in healthy children. The abundance of oral commensal species *Mycoplasma pneumoniae pneumonia, Streptococcus* spp., *Prevotella salivae* decreased in influenza patients.

These studies have identified specific bacterial species and community types associated with decreased susceptibility to influenza, indicating that targeting the microbiome could be a promising approach for preventing and managing influenza infections. The additional resolution acquired by whole genome metagenomic allowed further molecular characterization of the microbiota which can be useful for clinical interpretation. Further research is needed to better understand the causal relationships and develop effective interventions based on the respiratory microbiome.

#### HIV

Oropharyngeal swabs from children living with HIV in an orphanage in Bangalore were collected to determine bacterial composition by Illumina sequencing of the 16S rRNA partial genes (V3-V4). The genera *Proteus*, *Enterococcus*, *Bacteroides*, *Prevotella*, and *Clostridium* were the most prevalent bacterial populations in the oropharynx of the HIV positive patients (Ahmed et al., 2020). In another study Fukui et al. (2018) conducted a cross-sectional study to characterize the tonsil microbiome in 46 HIV-infected and 20 HIV-uninfected individuals from Toho University Omori Medical Center in Japan between October 2016 and June 2017. Analysis of the bacteriome and mycobiome by amplicon sequencing using Illumina MiSeq revealed that the palatine tonsil bacteriome of HIV-uninfected individuals differed from that of HIV-infected individuals in terms of relative abundance of *Neisseria* and *Haemophilus*. According to these authors, *Capnocytophaga ochracea*, *Neisseria cinerea*, and *Selenomonas noxia* were more abundant in the HIV-infected group than in the HIV-uninfected group (Fukui et al., 2018). Interestingly, fungal diversity and composition did not differ significantly between the two groups while microbial intercorrelation analysis revealed that *Candida* and *Neisseria* were negatively correlated with each other in the HIV-infected group (Fukui et al., 2018).

We identified two studies discussing microbiome and HIV, both used amplicon sequencing (16S) and targeted V4-V5 regions. One of the studies identified that there were some genera that where more abundant in HIV patients (*Neisseria* and *Haemophilus*) but found that there was no changed in fungal diversity using another amplicon sequencing targeting the ITS 1 rDNA gene). This is of interest as the most common secondary infections threatening HIV patients’ lives are usually caused by fungus such as Candida (Patil et al., 2018). People living with HIV are also known to be at higher risk of contracting bacterial *meningitis* and therefore the increased abundance of *Neisseria* and *Haemophilus* might point toward an explanation (van Veen et al., 2016), however more resolution to species level is necessary to identify if this increased abundance is driven by the pathogenic strains or only by the commensal harmless ones. The second study did not include a control group, which limits the ability to compare the results to those of healthy individuals and can only serve has a baseline information.

#### COVID-19

Covid-19, caused by the coronavirus SARS-CoV-2, is a global pandemic that is causing severe morbidity and mortality, as well as unprecedented economic and social disruption. Since it first detection in Wuhan, China in 2019, the rate of infectivity and mortality due to SARS-CoV-2 has incessantly increased over time, with count reaching approximately 767 million cases including 6. 9 million deaths globally as reported by WHO on May 3thrd 2023 (WHO Coronavirus (COVID-19) Dashboard, 2022). Clinical severity is variable among infected individuals depending on age, gender, body mass index, prior comorbidities, immune responses and genetics. The human oral microbiome is the second largest microbial community after the gut and may influence the onset and progression of several localized and systemic diseases, including those of viral origin, particularly for viruses that enter the body via the oropharynx. The human pandemic coronavirus SARS-CoV-2, which causes Covid-19, is one of several respiratory viruses for which the oropharynx is the main site of replication.

van Veen et al. (2016) conducted a cross-sectional clinical study analyzing oropharyngeal microbial metagenomes in healthy adults, patients with non-SARS-CoV-2 infections, or with mild, moderate and severe Covid-19 from seven German medical centers. This study used 16S rRNA gene sequencing and involved 345 participants to assess the role of the oropharyngeal microbiome in Covid-19 infection. A significant reduction in microbiome diversity and high dysbiosis of the upper respiratory microbiome were observed in hospitalized patients with severe Covid-19; they also observed that the abundance of *Haemophilus* or *Streptococcus* species in these oropharyngeal samples was predominant. These results provide insight into the role of the oropharyngeal microbiome in SARS-CoV-2 infection and may suggest new biomarkers for the severity of Covid-19 infection (de Castilhos et al., 2021).

Merenstein et al. (2021) also examined 507 oropharyngeal, nasopharyngeal and endotracheal samples of 83 hospitalized Covid-19 patients, along with non-Covid patients and healthy controls from the hospital of the University of Pennsylvania beginning in March 23, 2020, continuing through the first wave of the epidemic, and ending July 10, 2020. The bacterial communities in nasopharyngeal, oropharyngeal and bronchoalveolar lavages were determined by sequencing of 16S rRNA gene (V1-V2 region) using the Roche 454GS-FLX platform which yields comparable results to Illumina data. The observed diversity was inversely correlated with disease severity during hospitalization, lower lymphocyte-to-neutrophil ratio (LNR) was associated with both lower diversity (FDR = 0.03) and composition of the oropharyngeal microbiome (Merenstein et al., 2021). Common component of the human virome *Anelloviridae* and *Redondoviridae* showed higher colonization rates in severe disease cases. Among Covid-19 patients, abundance of *Proteobacteria* and *Actinobacteria* decreased in the oropharynx and was correlated with greater Covid-19 disease severity based on WHO score over the course of hospitalization (FDR = 0.008 and 0.05 respectively). At the genus level, patients with more severe disease had significantly lower relative abundances of *Hemophilus*, *Actinomyces*, and *Neisseria* (FDR < 0.05), all of which are abundant in the normal oropharyngeal microbiome. The airway microbiome and commensal virome were disrupted in Covid-19, correlating with a decrease in systemic immune parameters (Merenstein et al., 2021).

Ma et al. (2021) analyzed in China, the oropharyngeal microbiome of 31 confirmed patients with Covid-19, 29 flu patients with influenza B, and 28 healthy controls by shotgun metagenomic sequencing. One Covid-19 patient, all flu patients and healthy controls were admitted to Heilongjiang Provincial Hospital from January 20th to February 25th, 2020, and 30 Covid-19 patients admitted to Suihua First Hospital and Suihua Cancer Hospital from January 24th to February 25th, 2020. An increased abundance of *Klebsiella* sp., *Acinetobacter* sp. and *Serratia* sp. were associated with both disease severity and increased markers of systemic inflammation (neutrophil-lymphocyte ratio) (Ma et al., 2021). These results suggest that alterations in the oropharyngeal microbiota may have an impact on the severity of Covid-19 disease by influencing the inflammatory response leading to Covid-19 severity (Ma et al., 2021).

Rueca et al. (2021) recruited 39 individuals for their study from the end of February 2020 to the beginning of May 2020 in Italy; 21 patients had a laboratory-confirmed infection by SARS-CoV-2 from the National Institute for Infectious Diseases in Rome, Italy. They used 16S rDNA sequencing and observed a significant decrease in abundance of species when Covid-19 affected patients were compared to paucisymptomatic patients. *Deinococcus-Thermus* was only present in controls compared to SARS-CoV-2 patients admitted to intensive care, paucisymptomatic or affected by other coronaviruses (Rueca et al., 2021). *Candidatus Saccharibacteria* was significantly increased in negative controls and paucisymptomatic SARS-CoV-2 patients compared to patients admitted to intensive care for SARS-CoV-2. Complete depletion of *Bifidobacterium* and *Clostridium* was observed exclusively in SARS-CoV-2 Intensive Care Units (ICU) patients, which were also characterized by presence of *Salmonella*, *Scardovia*, *Serratia*, and *Pectobacteriaceae*. All these results showed that nasal/oropharyngeal microbiota profiles of SARS-CoV-2 patients can provide valuable information to facilitate patient stratification and pave the way for new therapeutic approaches to improve patient outcomes (Rueca et al., 2021).

Devi et al. (2022) studied nasopharyngeal and/or throat swabs collected at the MAX Hospital in Delhi, India. A total of 86 samples selected among 198 SARS-CoV-2 RT-PCR positive samples were studied to explore the presence of transcriptionally active microbes using Holo-Seq (Holo-transcriptome). The possible functional role of nasopharyngeal early resident transcriptionally active microbes in modulating disease severity was established, within groups of recovered patients with sub-phenotypes (mild, moderate, severe) and dead patients. An integrative approach combining patients’ clinical parameters, SARS-CoV-2 phylogenetic analysis, microbial differential composition, and their functional role have been used in this study. The clinical and phenotypes analysis led to the identification of transcriptionally active bacterial species associated with disease severity. They found significant transcript abundance of *Achromobacter xylosoxidans* and *Bacillus cereus* in the high mortality group, *Leptotrichia buccalis* in the severe group, *Veillonella parvula* in the moderate group, and *Actinomyces meyeri* and *Halomonas* sp. in the mild group of Covid-19 patients. The outcome of the study offers an opportunity to bring onward the less explored modulatory role of the microbiome alterations and disease severity in a hospitalized cohort of Covid-19 patients from India. Also, the presence of certain bacterial species associated with clinical groups provide leads for evaluating their probable roles in modulating the disease course in Covid-19 (Devi et al., 2022).

Gauthier et al. (2022) collected nasopharyngeal swab of SARS-CoV-2-infected and uninfected individuals during the Covid-19 pandemic in British Columbia, Canada from March 2020 to January 2022. They performed Nanopore full-length 16S rRNA sequencing on 194 nasopharyngeal swab specimens from hospitalized and community-dwelling SARS-CoV-2-infected and uninfected individuals. *Staphylococcus* was the most abundant genus in the two study groups not infected with SARS-CoV-2, whereas *Acinetobacter* was the most abundant genus in the hospitalized infected group and *Moraxella* the most abundant genus in the community-dwelling SARS-CoV-2 infected group. At the species level, both SARS-CoV-2-infected groups were dominated by common nasal pathobionts and opportunistic pathogens including *Haemophilus influenzae*, *Staphylococcus haemolyticus*, and *Staphylococcus aureus*, whereas SARS-CoV-2-infected hospitalized group had a majority of *Klebsiella aerogenes*, an opportunistic pathogen. At the family level, hospitalized patients infected with SARS-CoV-2 had a higher mean relative abundance and broader range of *Enterobacteriaceae* then all other study groups. The species *Cutibacterium acnes*, the genera *Cutibacterium* and *Peptinophilus*, and the families *Propionibacteriaceae* and *Peptostreptococcales-Tissierellales* were all differentially abundant over the four study groups (Kruskal–Wallis *p* < 0.05). An uncultured genus assigned to the family *Neisseriaceae* was also found to be differentially abundant. All the differentially abundant taxa were enriched in community-dwelling SARS-CoV-2 infected individuals. This study identified several changes in the nasopharyngeal microbiome associated with SARS-CoV-2 infection status and disease severity (Gauthier et al., 2022).

Children are less prone to severe disease and have milder illness courses than adults (Hurst et al., 2022). The factors underlying these age-associated differences are not well understood. The upper respiratory microbiome undergoes substantial shifts during childhood and is increasingly recognized as having an impact on host defense against respiratory infections. Thus, Hurst et al. (2022) sought to identify characteristics of the upper respiratory microbiome in relation to susceptibility to SARS-CoV-2 infection and its severity in relation to age. They collected clinical data and nasopharyngeal swabs from 285 children, adolescents, and young adults (<21 years) from Durham, North Carolina, United States with documented SARS-CoV-2 exposure. Illumina sequencing of 16S rRNA gene was used to characterize associations between nasopharyngeal microbiome characteristics and SARS-CoV-2 infection status and respiratory symptoms. These results revealed that the microbiota varied with age (PERMANOVA, *p* < 0.001; *R*^2^ = 0.06) and between SARS-CoV-2 infected individuals with and without respiratory symptoms (PERMANOVA, *p* = 0.002; *R*^2^ = 0.009). The SARS-CoV-2 infected participants with *Corynebacterium/Dolosigranulum*-dominant microbiome profiles were less likely to have respiratory symptoms than infected participants with other nasopharyngeal microbiome profiles. Moreover, nine bacterial taxa associated with SARS-CoV-2 infection and 6 taxa differentially abundant among SARS-CoV-2–infected participants with respiratory symptoms were identified; the magnitude of these associations was strongly influenced by age. The study allowed to identify links between age and specific nasopharyngeal microbiome features that are associated with SARS-CoV-2 infection susceptibility and symptoms in children, adolescents, and young adults. These data suggest that the upper respiratory microbiome may be a mechanism by which age influences SARS-CoV-2 susceptibility and illness severity (Hurst et al., 2022).

Similarly, Kim et al. (2022) examined associations between the salivary and nasopharyngeal microbiome and age, Covid-19 symptoms, and blood cytokines. The patients were part of a prospective observational cohort with Covid-19 related symptoms from Barnes-Jewish Hospital or affiliated Barnes-Jewish Hospital testing sites in Saint Louis, Missouri, USA, between March and September of 2020. A total of 78 saliva samples and 66 nasopharyngeal swabs from a Covid-19 patients’ cohort were collected and the V1-V2 region of the 16S rRNA gene characterized using Illumina MiSeq platform sequencing. They found that SARS-CoV-2 infection was associated with community-level differences in the oral and nasopharyngeal microbiomes. Salivary and nasopharyngeal microbiome alpha diversity negatively correlated with age and were associated with fever and diarrhea. Several bacterial genera were differentially abundant depending on the Covid-19 severity, including oral *Bifidobacterium*, *Lactobacillus*, and *Solobacterium*, all of which were reduced in patients with severe Covid-19. Nasopharyngeal *Paracoccus* was depleted while *Proteus*, *Cupravidus*, and *Lactobacillus* were increased in patients with severe Covid-19. Further analysis revealed that the abundance of oral *Bifidobacterium* was negatively related with plasma concentrations of known Covid-19 biomarkers interleukin 17F (IL-17F) and monocyte chemoattractant protein-1 (MCP-1). These results suggest that Covid-19 disease severity is associated with the relative abundance of certain bacterial taxa (Kim et al., 2022).

Seven studies explored the role of the pharyngeal microbiome in Covid-19 infection using either oropharyngeal or nasopharyngeal samples. Five studies used sequencing of 16S rRNA and the others employed shotgun metagenomic sequencing and complementary metagenomics sequencing approaches such as respiratory virus oligo panel (RVOP) and Holo-seq. Among the studies using 16S, two studies mentioned the use of the V1-V2 region. The shotgun metagenomic analyses allowed the characterization of the microbiota, beyond the simple identification of microorganisms; highlighting the enrichment in amino acid metabolism, xenobiotic biodegradation and detecting all 26 classes of antimicrobial resistance genes in the COVID-19 group, particularly in critical cases. Overall, these studies revealed that severe cases of Covid-19 were associated with a decrease in microbiome diversity and an imbalance in the microbiota composition (dysbiosis). Specific bacterial species, such as *Haemophilus*, *Streptococcus*, *Veillonella*, and *Megasphaera* were found to be more abundant in severe Covid-19 cases. These bacteria may serve as potential biomarkers for the severity of the infection. On the other hand, beneficial bacteria like *Actinomyces*, *Neisseria*, and *Rothia* were found to be reduced in severe cases. The studies also highlight the influence of the oropharyngeal microbiome on the immune response and inflammation. Dysbiosis of the microbiome was associated with local inflammation and a decrease in systemic immune parameters. Additionally, alterations in the oropharyngeal microbiota were linked to disease severity and increased markers of systemic inflammation. Age was found to be a factor affecting the composition of the nasopharyngeal microbiome and susceptibility to SARS-CoV-2 infection. Children, adolescents and young adults with specific nasopharyngeal microbiome profiles were less likely to exhibit respiratory symptoms when infected with SARS-CoV-2. In summary, these studies have shown that the oropharyngeal microbiome is influenced by COVID-19 infection and severity, highlighting the importance of understanding microbiome-virus interactions to gain valuable insights into disease progression and potential therapeutic strategies.

#### Influenza, parainfluenza, rhino, respiratory syncytial syndrome, corona, adeno, or metapneumo viruses

The bacterial content of the upper respiratory tract of asymptomatic healthy individuals and patients acutely infected with *influenza*, *parainfluenza*, *rhino*, *respiratory syncytial syndrome*, *corona*, *adeno,* or *metapneumo viruses* was determined through pyrosequencing of 16S rRNA targeting the V1 to V3 regions. A total of 59 patients with confirmed acute viral infections from Yonsei University Hospital in Seoul, Korea was selected in a study conducted by Yi et al. (2014) from December 2010 to May 2013. The study aimed to determine whether a viral infection-related bacterial profile exists in the respiratory tract and evaluate any disparities in the microbiota structure depending on the infectious virus species. This study showed that the oropharyngeal microbiome of healthy subjects were predominantly colonized by *Streptococcus,* while patients microbiota were enriched in *Haemophilus* or *Moraxella* (Yi et al., 2014). The *Moraxella nonliquefaciens* genome was found to encode various proteins capable of playing roles in pathogenesis (Yi et al., 2014). This study identified six types of oropharyngeal microbiome associated with the age of participants. No virus-specific bacterial profile was found, but comparative analysis identified *M. nonliquefaciens* as present in abundance in young infected patients (Yi et al., 2014). Overall, the bacterial composition of patients differed significantly from that of healthy individuals in terms of types and diversity.

### Oropharyngeal microbiome in bacterial diseases

The literature review found few studies that focused on the link between bacterial infectious diseases and alterations of the oropharyngeal microbiome. Among the diseases studied were pneumonia, meningitis, tuberculosis, and pharyngeal infections caused by *Neisseria gonorrhoeae*. Metagenomic techniques have allowed us to understand the ecology of the bacteria causing these diseases by characterizing several other microbial species that co-colonize the patients airways compared with healthy controls. These analyses allowed detection of significant microbiome variation in different stages of diseases providing useful data on the microorganisms present during disease onset and progression.

#### Pneumonia

Bacterial *pneumonia* is a major cause of morbidity and mortality worldwide, especially in immunologically vulnerable populations such as infants and the elderly. The pathogen responsible for most cases is *Streptococcus pneumoniae*. There was therefore a growing interest in understanding the link between the microbiome and *pneumonia*.

The oropharyngeal microbiome of elderly people with pneumonia was compared with that of healthy people in the Netherlands. This same comparison was made in young adults with pneumonia and asymptomatic controls using 16S RNA sequencing targeting the V5-V7 hypervariable region. The study showed a relatively high abundance of a number of microorganisms in the oropharynx, such as *Lactobacilli*, *Streptococcus (pseudo) pneumoniae*, and *Rothia*. As well as a lower abundance of several gram-negative *anaerobic bacteria*, including *Prevotella*, *Veillonella*, and *Leptotrichia* and the gram-positive genus *Parascardovia* in elderly patients with *pneumonia* compared to elderly controls (de Steenhuijsen Piters et al., 2016).

Furthermore, in the elderly, an aging immune system has been implicated in the increase of certain infections such as *pneumonia*. Whelan et al. (2014) investigated this by characterizing microbial communities in the upper respiratory tract of elderly population using 16S rRNA gene sequencing. These communities were compared to data from middle-aged adults. They showed that nasal and oropharyngeal microbiota present in adult populations can be reduced with age. The oropharynx and nasopharynx (OP/NP) were marked by a clear increase in relative streptococcal abundance with age. *Streptococcus* (26.1%), *Prevotella* (14.1%) and *Veillonella* (8.9%) dominated the pharynx of middle-aged population. Although the oropharynx of elderly individuals was also dominated by these three bacterial genera, *Streptococcus* had a significant increase in relative abundance (44.0%). This increase in *streptococci* includes pathogenic species, such as *Streptococcus pneumoniae*.

In another study by Pettigrew et al. (2016) on microbiome profiles in induced sputum samples and OP/NP samples, certain microorganisms were associated with the severity of community-acquired pneumonia in children. They demonstrated that in children from 6 months to less than 5 years of age, high relative abundance of *Actinomyces*, *Veillonella*, and *Rothia* in sputum specimens was associated with a reduced likelihood of 4-day hospitalization. High relative abundance of gram-negative aerobes *Haemophilus* and *Pasteurellacea* and low relative abundance of *Streptococcus* were associated with intensive care unit admission. High relative abundance of Gram-positive anaerobic *Gemella* species was associated with reduced likelihood of hospital stay ≥4 days in children aged 5 to 18 years. However, OP/NP specimen taxa were not associated with community-acquired pneumonia (CAP) severity in their study (Pettigrew et al., 2016).

### *Mycoplasma pneumoniae* pneumonia and ventilator-associated pneumonia

*Mycoplasma pneumoniae* frequently causes community-acquired pneumonia, affecting mainly children and adolescents. It is resulting in an increasingly high morbidity in Chinese children (Qin et al., 2014). In a study of 171 healthy children and 76 children with pneumonia, metagenomic sequencing targeting the V3-V4 region of 16S RNA was performed on oropharyngeal samples. The main oropharyngeal microbial species found in healthy children were *Prevotella* and *Streptococcus*. However, in children with *MPP*, oropharyngeal microbial diversity was decreased compared to healthy children. Thirty genus clusters accumulated significantly in the oropharyngeal microbiota of children with *MPP*, including 6 unknown and 24 known microbial species. These species were major respiratory pathogens such as *Mycoplasma pneumoniae, Staphylococcus epidermidis*, and *Staphylococcus aureus* (Dai et al., 2019).

In order to determine how imbalance of nasopharyngeal and oropharyngeal microbiota may be associated with lung microbiota in MPP, a study was conducted in Shenzhen Children’s Hospital in China by Dai et al. (2018). The study demonstrated that the respiratory microbiota of patients with pneumonia had a lower microbial diversity than that of healthy children. With potential transmission from nasopharyngeal (NP) microbiota to oropharyngeal (OP) and lungs, suggesting an association between respiratory microbiota imbalance and the severity of *Mycoplasma pneumoniae pneumonia*. The study also showed that a dominance of *Staphylococcus* in NP microbiota was associated with higher severity of acute respiratory infections (ARI), while a mixed NP microbiota was linked to a lower risk of ARI (Dai et al., 2018).

Similarly, Zhou et al. (2020) also demonstrated in a metagenomic study performed in China on children respiratory tract that *Streptococcus* was dominant in the oropharyngeal microbiota of healthy children with a prevalence of 30.47% (± 21.45). This prevalence increased to 34.79% (± 19.29) in MPP patients. There work aimed to further the understanding microbial biomarkers use in the prevention of *pneumonia*.

In order to determine whether the microbiome in tracheal and oropharyngeal secretions during intubation was associated with development of ventilator-associated pneumonia (VAP), a 16S rRNA gene-based culture and metataxonomic study was performed in mechanically ventilated adult patients at Geneva University Hospitals in Switzerland. They targeted the V1-V6 region of the bacterial 16S rRNA gene, followed by nested PCR and sequencing of the V3-V4 region. They found that a lower abundance of *Bacilli*, a normal Gram-positive of the oropharyngeal flora, in oropharyngeal secretions was associated with the development of late VAP with sensitivity and specificity above 80%. *Mycoplasma* was also frequently detected in respiratory specimens obtained during intubation in patients with VAP. These results may contribute to improved diagnosis and prevention of ventilator-associated *pneumonia* in the future (Emonet et al., 2019).

Sommerstein et al. also investigated the longitudinal dynamics of the oropharyngeal and tracheal microbiota in a cohort of mechanically ventilated patients at Bern University Hospital, Switzerland by comparing them to controls (Sommerstein et al., 2019). A total of 71 oropharyngeal swabs were collected from five VAP patients and five controls, respectively in their study. They demonstrated that the causative pathogen in three patients was an Enterobacteriaceae and two *Haemophilus influenzae*. Their characterization of the microbiome by 16S rRNA sequencing demonstrated that patients with *enterobacterial VAP* appear to develop lower intra diversity (alpha-diversity) of the oropharyngeal microbiota between the second and fifth day of mechanical ventilation, compared with patients with *Haemophilus influenzae* VAP. It is important to note that the detection of *Enterobacteriaceae* in the oropharynx occurred on the second day of follow-up and consisted of a single operational taxonomic unit in two-thirds of patients with *enterobacterial VAP*.

#### Secondary bacterial pneumonia

Recent studies have highlighted the clinical importance of influenza-associated bacterial pneumonia, but most have relied directly on the host immune response. Lu et al. (2017) characterized the oropharyngeal microbiome of *avian influenza A* (H7N9) patients with and without secondary bacterial lung infection, as well as healthy controls (HCs), using 16S rRNA-based sequencing analysis. The study was conducted at the First Affiliated Hospital, College of Medicine of Zhejiang University, Hangzhou, China.

They showed that compared to healthy patients, the degree of dysbiosis of the oropharyngeal microbiota in patients with avian influenza A (H7N9) and secondary bacterial lung infection (SBLI) was more severe than in the H7N9 group without SBLI. Indeed, opportunistic pathogenic genera, including *Streptococcus, Actinomyces, Rothia, Eubacterium, Oribacterium*, and *Mogibacterium* were more abundant in the microbiomes of H7N9_SBLI patients than in those of H7N9 patients. This study shows that the pharyngeal microbiome could provide new biomarkers for SBLI prevention.

We found nine studies that examined the link between the oropharyngeal microbiome and pneumonia. Most of these studies used 16S rRNA sequencing targeting the V3-V4 region. With the exception of Steenhuijsen Piters et al. who targeted the V5-V7 region. These studies suggest that pneumonia can be considered a polymicrobial disease, rather than being caused by a single pathogen. Indeed, an association between different bacteria present in the oropharyngeal/nasopharyngeal microbiome and the severity of pneumonia has been observed. Certain types of bacteria, such as *Streptococcus, Haemophilus*, *Pasteurellacea*, and *Prevotella*, appear to be associated with a more severe course of the disease in hospitalized children. Commensals bacteria, may play a role in modifying immune responses or influencing pathogen virulence. Furthermore, these results show that the oropharyngeal microbiota in children with *PPM* has a simpler structure compared to healthy children. This simplification is associated with *M. pneumoniae*’s direct competition with other bacterial species. Finally, *H7N9 avian influenza* patients who developed secondary pneumonia infections showed alterations in their oropharyngeal microbiota. Taken together, these studies underline the importance of the oropharyngeal microbiome in *pneumonia* and reveal new avenues of research to better understand and treat that infectious disease.

### Meningitis

*Neisseria meningitidis* is a commensal bacterium of the oropharyngeal cavity and can also causes bacterial meningitis. However, the impact of the oropharyngeal microbiome on meningococcal carriage has been largely unexplored. Here we highlight a whole genome metagenomic study of the oropharyngeal microbiome of 158 students following a meningococcal outbreak at a United States college to identify interactions between bacterial community composition and *N. meningitidis* carriage. The study showed that *Neisseria meningitidis* abundance was positively correlated with that of *Fusobacterium nucleatum*, consistent with the propionic acid cross-feeding hypothesis. Indeed, *N. meningitidis* is able to use propionic acid as a carbon source and can therefore benefit from cross-feeding with propionic acid producers such as *Fusobacterium nucleatum* under nutrient-poor growth conditions (Catenazzi et al., 2014). Other species showed positive abundance correlations with *Neisseria meningitidis*, including *Aggregatibacter aphrophilus*, *Campylobacter rectus, Catonella morbi, Haemophilus haemolyticus*, and *Parvimonas micra*. The abundance of *N. meningitidis* was negatively correlated with unidentified *Veillonella* species (Retchless et al., 2020). These studies suggest that *N. meningitidis* finds a favorable environment in the pharynx, where other bacteria produce propionic acid. Complex interactions between different bacterial species in the oropharynx may influence colonization by *N. meningitidis*.

#### Sexually transmitted pharyngeal infections

The oropharynx represents a potential site of residence for *Neisseria gonorrhoeae*. It is a suitable ecological niche for the bacteria to reproduce and persist over time. The composition of the oropharyngeal microbiome of 70 swabs from the S. Orsola-Malpighi Hospital in Bologna (Italy) was analyzed by comparing uninfected subjects with patients with pharyngeal gonorrhea by sequencing of the V3-V4 hypervariable regions of the 16S rRNA gene by Marangoni et al. (2020). They showed that the pharyngeal microbiome of *Neisseria gonorrhoeae* positive individuals showed an abundance of several anaerobes such as *Treponema, Parvimonas, Peptococcus, Catonella, Filifactor* and were reduced in various aerobic genera such as *Pseudomonas* and *Escherichia,* compared to uninfected controls (Marangoni et al., 2020).

As the incidence of resistant gonorrhea increases, the likelihood of the spread of antimicrobial resistance increases and men who have sex with men (MSM) are disproportionately affected. Hence, the use of antiseptic mouthwashes has been proposed as an intervention to reduce transmission. With this in mind, Plummer and colleagues studied the effect of 12 weeks of mouthwash use on oropharyngeal microbiota using 16S rRNA gene sequencing in participants in Australia. This study showed a small but significant decrease in the abundance of *Streptococcus* and *Leptotrichia* after the use of Biotene, a brand of mouthwash. This result attests that daily use of antiseptic mouthwashes may have minimal long-term effects on the composition of the oropharyngeal microbiome (Plummer et al., 2022).

These results highlight the importance of understanding the interactions between the microbiome and *Neisseria gonorrhoeae* infection in the pharyngeal and oral environments and suggest potential strategies for prediction and prevention of pharyngeal *gonorrhea*.

### Tuberculosis

Tuberculosis is a respiratory disease caused by *Mycobacterium tuberculosis* and is responsible for many deaths and morbidity worldwide each year (Global Tuberculosis Reports, 2022).

Sputum, oropharyngeal and nasal airway samples were collected from tuberculosis patients and healthy individuals in Colombia, followed by microbiome characterization using metagenomic technique targeting the V1-V2 hypervariable region of 16S rRNA. The majority of sequences in all samples from tuberculosis patients and healthy controls belonged to five phyla: *Firmicutes*, *Bacteroidetes*, *Proteobacteria*, *Actinobacteria*, and *Fusobacteria*. The only difference between the patients and controls groups was found in the oropharyngeal samples, where unclassified sequences belonging to the family *Streptococcaceae* were more abundant in the TB patients.

These results show that he oropharynx is an appropriate site to study differences in the airways microbiome between TB patients and health individual (Botero et al., 2014). This study provides interesting information for understanding a potential role of the microbiome in the pathogenesis of this bacterial diseases.

## Discussion and conclusions

This literature review showed that different parts of the human body harbor specific microbial populations and that certain microbial profiles are associated with emergence of different pathologies. The oropharyngeal microbiome, despite its importance, remains little studied and is often sampled in combination with other oral sites, especially the nasopharynx. This literature review identified a limited number of articles on the link between the pharyngeal microbiome and certain bacterial and viral infections. This number has increased exponentially regarding viruses since 2020 due to research conducted on the Covid-19 pandemic using oropharyngeal and nasopharyngeal samples.

The current research shows that the study of the microbiome can be useful in the biomedical field. The use of metagenomic, an unbiased method for microorganisms’ identification, has improved the resolution at which the microbiota can be studied. Most studies have focused on the identification of bacteria present through the use of 16S RNA sequencing and only few studies used whole genome sequencing metagenomic that can go further to study the downstream mechanistic aspects. However, several papers identified the potential use of certain bacteria as biomarkers of various infections based on their abundance in specific diseases states compared to healthy controls. The identification of these specific biomarkers can be useful for development of diagnostic tools, these results also suggests that modulation of the microbiome could help maintain health or prevent various diseases in addition to antibiotic therapy and vaccination. Determining how the microbiome responds to treatments, diets or disease states could also potentially foster more targeted treatments.

However, further research is needed to better understand the complex molecular mechanisms involved in these processes and especially establish a causal relationship between the presence of a particular microorganism and the development or prevention of a disease before effective treatment with prebiotics, probiotics and live biotherapies can be envisioned. Beside the larger application of whole genome sequencing metagenomic, these could also involve *in vitro* and *in vivo* assessment of the importance of identified microorganisms in the oropharyngeal ecological niche and proteomic analysis to determine downstream effect on the biochemical pathways.

Indeed, the different articles obtained in this review show that the results are sometimes different from one metagenomic study to another. This can be due to the different methodology (extraction kits, variable region chosen, metagenomic approaches) and samples type used (Farraj et al., 2020). The difference could also be due to factors that influence the variation of microbial diversity such as differences in climate, nutrition style or certain environmental factors (Catania et al., 2021; Panthee et al., 2022). More standardized approaches are needed to better understand the role of the microbiome in multiple aspects of health and to allow better comparison between studies. For example, a revision, by a diverse and representative microbiome research community, of “the human microbiome project” (Turnbaugh et al., 2007) standardized protocols and rigorous application of the updated versions would allow implementation of agreed best practices in sampling, sequencing methodologies (16S primers for example) and data analysis relevant to different body sites and research questions. Regular assessment and update of the data analysis pipelines will also be needed to keep up with a rapidly evolving bioinformatic field.

Most articles identified refer to studies conducted in relatively high-income countries (United States, United Kingdom, Europe, China, Japan) and very little data from Africa and East Asia have been detected using our search criteria which were specific to the oropharyngeal microbiome and infectious diseases using metagenomic approaches. However, other review articles have highlighted the important differences to consider when studying the microbiome from relatively understudied populations such as in Africa or Southeast Asia (Chotirmall et al., 2017). More studies are needed in those population to have a more global picture of the microbiome in general and the oropharyngeal microbiome in particular.

The current review focused on the most recent results that evaluated the composition of the pharyngeal microbiome in viral and bacterial infections using metagenomic methodologies. Most of the studies focused on the bacterial microbiome which represent only a portion of the human microbiomes. Study of the interactions of all part of the microbiome including bacteria, virus and archaea, would also be necessary to have a more realistic and complete understanding of the role of the microbiome in the development of bacterial and viral diseases. Integration of other omics, such as RNA sequencing, or metatranscriptomics would provide direct information on the expression of active genes to understand the metabolic profile of the microbiome (Shakya et al., 2019).

## Author contributions

KD: Conceptualization, Data curation, Formal analysis, Funding acquisition, Methodology, Supervision, Validation, Writing – original draft, Writing – review & editing. KM: Data curation, Formal analysis, Investigation, Methodology, Writing – original draft, Writing – review & editing. JT: Data curation, Formal analysis, Investigation, Methodology, Writing – original draft, Writing – review & editing. TA: Supervision, Validation, Writing – review & editing. BKB: Supervision, Validation, Writing – review & editing. BB: Conceptualization, Funding acquisition, Project administration, Supervision, Validation, Writing – review & editing.
